# Fear Conditioning in an Abdominal Pain Model: Neural Responses during Associative Learning and Extinction in Healthy Subjects

**DOI:** 10.1371/journal.pone.0051149

**Published:** 2013-02-26

**Authors:** Joswin Kattoor, Elke R. Gizewski, Vassilios Kotsis, Sven Benson, Carolin Gramsch, Nina Theysohn, Stefan Maderwald, Michael Forsting, Manfred Schedlowski, Sigrid Elsenbruch

**Affiliations:** 1 Inst. of Medical Psychology & Behavioral Immunobiology, University Hospital of Essen, University of Duisburg-Essen, Essen, Germany; 2 Clinic of Neuroradiology, Innsbruck Medical University, Innsbruck, Austria; 3 Institute of Diagnostic and Interventional Radiology and Neuroradiology, University Hospital of Essen, University of Duisburg-Essen, Essen, Germany; 4 Erwin L. Hahn Institute for Resonance Imaging, University of Duisburg-Essen, Essen, Germany; Tokai University, Japan

## Abstract

Fear conditioning is relevant for elucidating the pathophysiology of anxiety, but may also be useful in the context of chronic pain syndromes which often overlap with anxiety. Thus far, no fear conditioning studies have employed aversive visceral stimuli from the lower gastrointestinal tract. Therefore, we implemented a fear conditioning paradigm to analyze the conditioned response to rectal pain stimuli using fMRI during associative learning, extinction and reinstatement.

In N = 21 healthy humans, visual conditioned stimuli (CS^+^) were paired with painful rectal distensions as unconditioned stimuli (US), while different visual stimuli (CS^−^) were presented without US. During extinction, all CSs were presented without US, whereas during reinstatement, a single, unpaired US was presented. In region-of-interest analyses, conditioned anticipatory neural activation was assessed along with perceived CS-US contingency and CS unpleasantness.

Fear conditioning resulted in significant contingency awareness and valence change, i.e., learned unpleasantness of a previously neutral stimulus. This was paralleled by anticipatory activation of the anterior cingulate cortex, the somatosensory cortex and precuneus (all during early acquisition) and the amygdala (late acquisition) in response to the CS^+^. During extinction, anticipatory activation of the dorsolateral prefrontal cortex to the CS^−^ was observed. In the reinstatement phase, a tendency for parahippocampal activation was found.

Fear conditioning with rectal pain stimuli is feasible and leads to learned unpleasantness of previously neutral stimuli. Within the brain, conditioned anticipatory activations are seen in core areas of the central fear network including the amygdala and the anterior cingulate cortex. During extinction, conditioned responses quickly disappear, and learning of new predictive cue properties is paralleled by prefrontal activation. A tendency for parahippocampal activation during reinstatement could indicate a reactivation of the old memory trace. Together, these findings contribute to our understanding of aversive visceral learning and memory processes relevant to the pathophysiology of chronic abdominal pain.

## Introduction

Although fear conditioning is well-established to elucidate the mechanisms mediating associative learning and memory processes [1], [2], very little is known in the context of visceral pain. In humans, Pavlovian conditioning with aversive visceral unconditioned stimuli (US) has been accomplished in conditioned nausea and vomiting [3], [4] and the role of interoceptive fear conditioning in chronic pain has been proposed [5]. However, fear conditioning with aversive *visceral* stimuli as US has not been accomplished thus far, with the exception of a single esophageal distension study [6].

It is important to complement and extend these findings using aversive US from the *lower* gastrointestinal tract, i.e., rectal distensions, for several reasons. First, from an evolutionary view, the ability to discriminate and associate situations or cues that predict the occurrence of abdominal pain is extremely important to allow effective (survival) strategies, such as avoidance of specific foods or contexts indicating the presence of bacterial or viral challenges. Hence, associative learning involving aversive visceral US is almost certainly preserved across species and can be considered a fundamental learning process that remains poorly studied. Furthermore, there is evidence to support that associative learning processes could be important in the aetiology of clinical conditions associated with chronic abdominal pain and/or visceral hyperalgesia. For example, learned associations between predictive contextual cues and painful stimuli were reportedly relevant for the development of visceral hypersensitivity [7] and for the retrieval of visceral pain-conditioned passive avoidance in rats [8]. In irritable bowel syndrome (IBS), “conditioning” led to reduced pain thresholds [9] and pain-predominance correlated with the development of rectal hypersensitivity after a noxious sigmoid “conditioning” stimulus [10]. Finally, classical trace eyeblink conditioning was altered in fibromyalgia [11], a condition which often overlaps with IBS [12].

However, it is the important role of anxiety (and possibly of fear) in the pathophysiology of IBS that most strongly supports the relevance of fear conditioning with visceral stimuli. While anxiety is defined as the anticipation of a *potential* (future) threat, fear constitutes an adaptive response to *immediate* threat and is hence a time-limited response to actual adversity [13]. Consequently, it is fear (rather than anxiety) that is studied using Pavlovian fear conditioning paradigms. Nevertheless, it is unequivocal that fear conditioning studies provide important knowledge about various aspects of anxiety disorders [14]–[16]. The overlap between IBS (and other somatization disorders) with pre-clinical as well as clinical anxiety [17] is well-established, and the role of anxiety in central visceral pain processing is increasingly appreciated [18]. Fear, on the other hand, is an aspect that has previously not been systematically considered, but may be just as relevant.

Although a number of brain imaging studies (using somatic or auditory US) have elucidated the neural mechanisms mediating conditioned fear [1], only the above mentioned single imaging study employed aversive esophageal distensions as US [6]. Therefore, we implemented the first fear conditioning study in which the conditioned neural anticipatory response to rectal pain stimuli was analyzed with fMRI in healthy subjects. In addition to assessing the associative learning process, we also aimed to study aspects of fear memory by including not only an extinction but also a reinstatement phase. Extinction is conceptualized as a form of new inhibitory learning rather than simply as “forgetting” or “erasing” of an old memory [19]. This view of extinction resulted from experimental findings demonstrating “spontaneous recovery” of a previously extinguished memory after a delay as well as of the application of different retrieval techniques including “renewal” and “reinstatement” paradigms [20]–[22]. The “renewal-effect” describes the return of conditioned responses to the conditioned stimulus (CS) after a change of context following extinction. “Reinstatement” is defined as the retrieval of an extinguished memory after unexpected and unpaired exposure to the US. Both techniques provide important tools into the mechanisms of memory consolidation and reconsolidation [20]–[22]. Given previous human studies addressing reinstatement in the context of fear conditioning (reviewed in [16]), herein, we chose to implement a reinstatement procedure as a first step.

We aimed to test the following specific hypotheses: (1) The acquisition of conditioned anticipatory fear in response to a previously neutral conditioned stimulus (CS) is mediated by the central fear network, as evidenced by activation of the amygdala, but also involves structures participating in the processing of the conditioned and unconditioned stimuli, namely the anterior cingulate cortex, somatosensory cortex, precuneus and insula. (2) Extinction constitutes a learning process regarding new predictive cue properties, which is mediated by the hippocampus and prefrontal cortex. (3) Reinstatement involves activation of the hippocampus, amygdala and prefrontal cortex, based on previous studies demonstrating participation of the hippocampus (human studies) [21], [23] and of connections between the amygdala and prefrontal cortex (animal studies) [24].

## Methods

### Ethics statement

The study protocol was approved by the local Ethics Committee (University Hospital of Essen, University of Duisburg-Essen, Germany) and follows the rules stated in the Declaration of Helsinki. All participants gave written informed consent and were paid for their participation.

### Inclusion and exclusion criteria

Twenty-one healthy males and females were recruited by local advertisement. General exclusion criteria included age<18 years and >45 years, body mass index <18 or >27, any concurrent medical condition, including neurological, psychiatric, cardiovascular, immunological and endocrine conditions, evidence of structural brain abnormality upon structural MRI scan, and the usual MRI-specific exclusion criteria (i.e., phobic anxiety, claustrophobia, ferromagnetic implantations). Only females on oral contraceptives were included to reduce potential confounding by menstrual cycle phase. All participants were evaluated digitally for anal tissue damage (e.g., painful haemorrhoids) which may interfere with balloon placement. A history of psychological conditions (based on self-report) or presently increased scores on the Hospital Anxiety and Depression Scale (HADS) [25] were also exclusionary. Frequency and severity of gastrointestinal complaints suggestive of any functional or organic gastrointestinal condition were assessed with a standardized in-house questionnaire. Pregnancy was excluded by commercially available urinary test on the day of the study.

### Study design

Each subject completed the study protocol (total duration approximately two hours) on one study day. The time of day was not standardized due to irregular availability of scanner time. Upon arrival at the laboratory, rectal perceptual and pain thresholds were initially determined outside the scanner using established methodology (see below, duration approximately 25 minutes). Following a rest period of 10 minutes, a structural MRI scan was then completed to exclude brain abnormalities and to familiarize subjects with the scanner environment (duration approximately 5 minutes). Third, using event-related fMRI, brain activation was measured during the anticipation and delivery of visceral stimuli in a within-subject, repeated measures design with three separate, consecutive scanning sessions, i.e., acquisition, extinction, reinstatement (for details, see conditioning protocol, total duration of all three fMRI sessions: 53.08 minutes). In all sessions, visual conditioned stimuli (CS) or a visual control stimulus (“frame”) were presented. As unconditioned stimuli (US), rectal distensions just below the individual pain threshold were employed. At the conclusion of each session, subjects were prompted to complete ratings of CS-pleasantness and unpleasantness, perceived CS-US contingency and present tension.

**Figure 1:**
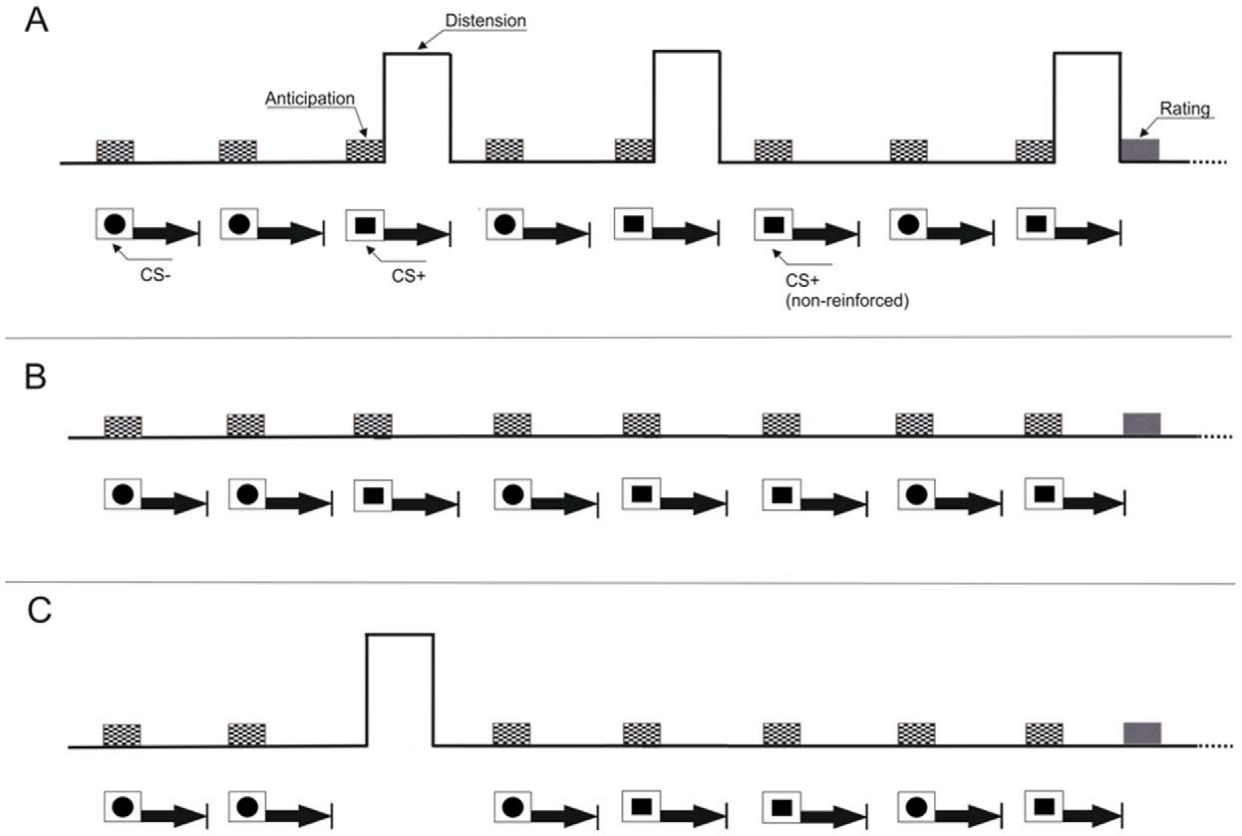
Study design. Study design illustrating the conditioning protocol consisting of an acquisition phase (A) during which painful rectal distensions were paired with a visual CS^+^ while a second visual stimulus (CS^−^) was presented without US (differential conditioning using geometric symbols as CSs). A total of 32 CSs were presented (16 CS^+^; 16 CS^−^) in pseudo-randomized order. Twelve out of the 16 CS^+^ were followed by a US (i.e., 75% reinforcement schedule). The onset of the US presentation varied randomly between 7.2 and 12s after CS^+^ onset, and both stimuli were co-terminated (i.e., delay conditioning). In the extinction phase (B), CSs (12 CS^+^; 12 CS^−^) were presented without US. In the reinstatement phase (C), one single unpaired US was delivered during the initial off-phase. Subsequently, only CSs (6 CS^+^; 6CS^−^), were presented. At the end of each phase, online ratings assessing CS unpleasantness were accomplished. In addition, at the end of the acquisition and reinstatement phases, perceived CS-US contingency ratings together with distension-induced pain and current tension ratings were accomplished.

The conditioned anticipatory blood-oxygen-level-dependent (BOLD) response was quantified using data from scans from CS onset until US onset in order to separate anticipatory activation from distension-related activation, as illustrated in Fig. 1. To address the primary hypotheses, the contrasts CS^+^>CS^−^ and CS^+^<CS^−^ were analyzed. Additionally, distension-induced activation, averaged over all delivered distensions, was analyzed to confirm visceral pain-associated neural activation in this paradigm (for details, see “fMRI imaging and analyses”).

### Conditioning protocol

We chose to implement a delay conditioning protocol which involves presentation of a CS^+^ that overlaps with presentation of the US rather than a trace conditioning paradigm which is characterized by a separation between presentation of the CS^+^ and presentation of the US. Delay conditioning paradigms are more common in human fMRI studies, especially in studies focusing on extinction processes, and lead to more rapid acquisition and faster extinction which we considered a relevant advantage given a relatively long fMRI study protocol of 53 minutes in our case (for details, see below) [1]. Furthermore, we decided against inclusion of a habituation phase (with presentations of the US alone) based on the following considerations: First, we considered the rectal sensory and pain threshold assessments together with the structural MRI (both completed just prior to the fMRI study) as a habituation phase where subjects were familiarized both with the distensions and the MR environment. Second, we neither wanted to further increase the number of painful rectal distensions delivered or increase the total amount of time subjects had to spend in the MR scanner given that the total duration of the fMRI study alone was 53 minutes.

In the acquisition phase ( Fig. 1A , duration 24.58 minutes), painful rectal distensions (US; duration 16.8 seconds) were paired with a visual CS^+^ while a second visual stimulus (CS^−^) was presented without US (differential conditioning). White geometric symbols (i.e., a circle, a square) were chosen as visual CS. Half the subjects received squares as CS^+^ and circles as CS^−^ and vice versa. All CSs were presented within a white frame on a black background; during off-phases, only the frame was presented (visual control stimulus: “frame”). A total of 32 CSs were presented (16 CS^+^; 16 CS^−^) in pseudo-randomized order (maximum of 2 consecutive identical CSs). Twelve out of the 16 CS^+^ were followed by a US (i.e., 75% reinforcement schedule). This schedule was chosen based on previous evidence that an unpredictable US is more aversive, increases fear responses along with enhanced activation of fear-related brain regions, reviewed in [1]. The onset of the US presentation varied randomly between 7.2 s and 12 s after CS+ onset, and both stimuli were co-terminated (i.e., delay conditioning). This variable jittering image acquisition technique was chosen to improve spatial and temporal resolution [26]. Inter-trial intervals (ITI) were 20 s.

In the extinction phase ( Fig. 1B , duration 17.89 minutes), only CSs (12 CS^+^; 12 CS^−^) in same order as in the acquisition phase were presented without US. Experimentally, reinstatement is defined as the retrieval of a formerly extinguished memory after unexpected and unpaired exposure to the unconditioned stimulus. Accordingly, in the reinstatement phase ( Fig. 1C , duration 10.26 minutes), one single unpaired US was delivered during the initial off-phase. Subsequently, only CSs (6 CS^+^; 6CS^−^), were presented without US, again in the same order as was accomplished in the other phases. The total duration of all three fMRI session was 53.08 minutes. There were no breaks in-between sessions other than the online ratings that were accomplished at the conclusion of each session. All stimuli and online ratings (see below) were presented using commercially available stimulus delivery and experimental control software (Presentation®, Neurobehavioral Systems, Albany, CA, U.S.A.).

### Online ratings

**Figure 2:**
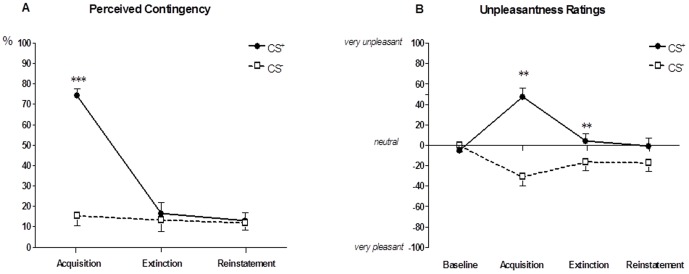
Online ratings. Perceived CS-US contingency (A) and CS unpleasantness (B) assessed separately for CS^+^ and CS^−^ using online visual analogue scales. For contingency, the ANOVA interaction was significant (time X type of CS: p = .0001) and post-hoc tests revealed significantly greater perceived CS^+^-US contingency following the acquisition phase when compared to perceived CS^−^-US contingency (***p = .0001). For unpleasantness, there was also a significant ANOVA interaction (time X type of CS: p = .0001), and post-hoc tests showed significantly greater CS^+^ unpleasantness following the acquisition (**p = .001) and the extinction (**p = .006) phases. Data are shown as mean ± SEM.

Online ratings were accomplished using a MRI-compatible hand-held fiber optic response system with keypads that subjects were instructed to press in order to move a cursor up or down when prompted (LUMItouch™, Photon Control Inc., Burnaby, BC, Canada). Using this system, subjects rated perceived CS pleasantness/unpleasantness, perceived CS-US contingency, present-state tension, and distension-induced pain on visual analogue scales (VAS) prior to (for pleasantness/unpleasantness and tension) and additionally after each session (for pleasantness/unpleasantness, contingency, tension, pain). Specifically, subjects responded to the following questions: For pleasantness/unpleasantness: “How do you find the square/circle?” with the ends of this scale defined as “very pleasant” – “very unpleasant” (in the middle of the scale, the word “neutral” was shown). We included this rating of CS unpleasantness/pleasantness as one important indicator of fear conditioning to show that a previously neutral CS turned into an aversive CS following CS-US pairings. For perceived contingency, the specific question was: “How often was a square/circle followed by a painful stimulus” with ends of the scales defined as “never” – “always”. This contingency scale was included to assess aspects of cognitive awareness regarding CS-US associations. For tension, the specific question asked was: “How tense do you feel at this moment?” with ends defined as “not tense” – “very tense”. This scale was included to allow online assessments of overall tension (as an indicator of psychological stress levels) which could be relevant in follow-up studies addressing for example group differences in patients and controls where differential effects of tension/stress on the conditioning and/or pain procedures may be present. Additionally, perceived painfulness of distensions was assessed at the conclusion of the acquisition phase with the question: “How painful were the stimuli?” and at the conclusion of the reinstatement phase with the question: “How painful was the last stimulus?” with ends defined as “not painful” – “very painful”. This VAS scale was included as a control variable to ensure that the US was indeed adequately painful. For analysis, all responses were quantified in mm (or % for contingency) from “0” to “100”, except for the combined pleasantness/unpleasantness scale which was quantified from −100 to 100 mm (with 0 indicating “neutral”, see Fig. 2B). Note that although similar kinds of visual analogue scales are frequently used in experimental research and are considered a valid and psychometrically sound method of quantifying subjective ratings in human studies, these particular VAS scales as used herein have not been specifically validated.

### Rectal Distensions

Rectal distensions were carried out with a pressure-controlled barostat system (modified ISOBAR 3 device, G & J Electronics, Ontario, Canada). Briefly, perception and pain thresholds were determined outside the scanner using double-random staircase distensions with random pressure increments of 2–8 mmHg. Subjects were prompted to rate the sensation as follows: 1 = no perception, 2 = doubtful perception, 3 = sure perception, 4 = little discomfort, 5 = severe discomfort, still tolerable, 6 = pain, not tolerable. The threshold for first perception was defined as the pressure when the rating changed from 2 to 3; the pain threshold as the pressure at which the rating changed from 5 to 6. For repeated distensions during the fMRI study, distension pressures 2 mmHg below the individual pressure at which a rating of “6” was achieved. The maximal distension pressure was set at a pressure level of 50 mmHg.

### FMRI imaging and analyses

All MR images were acquired using a 3T MR (Skyra, Siemens Healthcare, Erlangen, Germany). 16 elements of the standard 20 channel head/neck DirectConnect coil were used. For structural images, a 3D-MPRage T1-weighted sequence (TR 1900 ms, TE 2.13 ms, TI 900 ms, flip angle 9°, FOV 239×239 mm^2^, 192 slices, slice-thickness 0.9 mm, voxel size 0,9×0,9×0,9 mm^3^, matrix 256×256 mm^2^, GRAPPA r = 2) was acquired. Blood oxygen level-dependent (BOLD) contrast images with fat saturation were acquired using echo-planar imaging (TR 2400 ms, TE 26 ms, flip angle 90°, FOV 240×240 mm^2^, and matrix 94×94 mm^2^, GRAPPA r = 2) with 42 transversal slices angulated in direction of the corpus callosum with a thickness of 3 mm and a 0.45 mm slice gap. Voxel-size was 2.6×2.6×3 mm^3^. For analysis, SPM 08 software (Wellcome Department of Cognitive Neurology, London, UK) was used. Prior to statistical analysis, images were realigned to the mean, normalized to a standard EPI-template as implemented in SPM8 and finally smoothed with an isotropic Gaussian kernel of 8 mm. Data were also subjected to high- and low-pass filtering and correction for temporal autocorrelations (based on a first-order autoregressive model).

Data analysis was performed using a general linear model (GLM) approach. All regressors were obtained by convolving a box-car function of the event duration with the canonical hemodynamic response function implemented in SPM. Specific effects were tested with appropriate linear contrasts of the parameter estimates for the different regressors resulting in a t-statistic for each voxel. After model estimation, the ensuing first-level contrast images from each subject (CS^+^>CS^−^; CS^+^<CS^−^ for all primary hypotheses) were computed on the first level and were then used for second-level analyses treating individual subjects as a random factor and including non-sphericity correction as follows:

- To verify previous imaging findings using rectal stimuli, neural activation in regions-of-interest (ROIs, see below) was analyzed to confirm pain-related neural activation irrespective of learning aspects. To do so, a one-sample t-test was computed for distension-induced activation for all N = 12 distensions delivered in the acquisition phase.
- To test our specific hypotheses, *anticipatory* conditioned BOLD responses were analyzed by computing the contrasts CS^+^>CS^−^ and CS^+^<CS^−^ (scans from CS onset until US onset) for each subject on the first level. Second-level analysis was then accomplished with one-sample t-tests to assess activation in specific ROIs for different experimental phase as follows: (1.) In the acquisition phase, the anticipatory conditioned response was quantified by determining the BOLD response in-between CS and US onsets for *early* (first 5 CS^+^, first 5 CS^−^) and *late* (last 5 CS^+^, last 5 CS^−^) acquisition separately given previous reports using similar methodology ^1^. Data from the intermediate period (6 CS^+^, 6 CS^−^) were omitted, except for complementary analyses and for a better visualization of amygdala activation (for details, see below). The correlation between amygdala peak voxel activation in the late acquisition phase and CS^+^ unpleasantness ratings was assessed by computing Pearson's r. (2.) In the extinction phase, a total of 24 CS (12 CS^+^; 12 CS^−^), were separated into an *early* (first 6 CS^+^; 6 CS^−^) and a *late* (last 6 CS^+^; 6 CS^−^) extinction phase. The duration (i.e., number of scans) for the CS^+^>CS^−^ and CS^+^<CS^−^ contrasts was identical to the acquisition phase (even though no US were presented herein). In both acquisition and extinction phases, all second-level analyses were computed with CS unpleasantness change as a covariate of interest, since we considered an increase in CS^+^ unpleasantness following the acquisition of conditioned fear and a decrease in CS^+^ unpleasantness following extinction constitutes the most suitable indicator of successful fear conditioning and extinction at the subjective level in our study.

(3.) In the reinstatement phase, a total of 12 CS (6 CS^+^; 6 CS^−^) were again contrasted as CS^+^>CS^−^ and CS^+^<CS^−^ on the first level. On the second level, one-sample t-tests were computed to describe activation in specific ROIs (see below). The neural activation was covaried with the perceived painfulness as a covariate of interest herein.

For a more refined understanding of amygdala responses to the CS^+^ and the CS^−^ over the course of the learning and extinction processes, we conducted the following complementary analyses: First, we computed separate one-sample t-tests contrasting CS^+^ and CS^−^ for early, intermediate and late acquisition. Second, for a better visualization of amygdala activation, we extracted, averaged, and plotted contrast estimates for all phases of the experiment.

Based on previous fMRI fear conditioning studies with aversive USs [1], ROIs included the amygdala, hippocampus, insula, somatosensory cortex, precuneus, anterior cingulate cortex, and prefrontal cortex [dorsolateral prefrontal cortex (DLPFC); ventrolateral prefrontal cortex (VLPFC), orbitofrontal cortex (OFC)]. ROI analyses were carried out using anatomical templates constructed from the WFU Pick Atlas (Version 2.5.2), as implemented in SPM [27], and applying familywise error (FWE) correction for multiple comparisons. For ROI analyses, we only report p<.05, indicated throughout the text as p_FWE_<.05. Given that this constitutes the first study using rectal pain stimuli as US, we additionally report results of exploratory analyses using a more liberal threshold of p<.001 (uncorrected). All results are given as MNI coordinates.

### Statistical analysis of online ratings and other non-fMRI data

VAS ratings were analyzed with repeated measures analysis of variance (ANOVA) with two repeated factors (time, type of CS) followed by paired-tests corrected for multiple comparisons using Bonferroni correction to compare individuals means. Correlations between subjective measures and neural activation with the amygdala were accomplished by computing Pearson's R. In all analyses, the alpha level for significance was set at .05 and results are shown as mean ± standard error of the mean (SEM).

## Results

### Participants

A total of 21 healthy subjects participated (71.4%, N = 15 male; 28.6%, N = 6 female); due to technical errors (N = 1) and movement artifacts (N = 1) full fMRI data sets were available for N = 19 subjects. Mean age was 24.06±2.70 years. Ninety-five % (N = 20) had completed the German high school level that allows entry of a University “Abitur”; 85.7% (N = 18) were non-smokers, and all had a normal weight (BMI 24.09±0.74). Anxiety and depression scores, assessed with the HADS, were low and well-within the normal range (mean ± SEM: 4.32±3.29 for depressions score; 2.06±2.67 for anxiety score). Mean sensory and pain thresholds were 16.57±0.99 mmHg and 35.43±2.22 mmHg, respectively.

### Subjective and neural responses to painful rectal distensions

To verify that rectal distensions indeed constituted an appropriately aversive visceral unconditioned stimulus, we assessed perceived pain and present state tension at several time points using online VAS ratings. For distension-induced pain, mean pain ratings were 73.31±4.34 mm at the conclusion of the acquisition phase (i.e., rating of the preceding 12 distensions) and 56.50±6.78 mm at the conclusion of the reinstatement phase (i.e., rating of only one single preceding distension). For tension ratings, the ANOVA revealed a significant time effect (F = 4.29, p = .008). Post-hoc tests demonstrated that tension increased significantly from baseline to the conclusion of the acquisition phase (baseline: 27.81±5.30 mm; acquisition phase: 48.38±6.12 mm, paired t-test: p = .007). Ratings remained high following the extinction phase (44.91±6.26 mm), but did no longer differ significantly from baseline ratings, and normalized following the reinstatement phase at the conclusion of the experiment (33.14±5.32 mm).

At the level of the brain, we analyzed neural responses to all 12 distensions delivered in the acquisition phase to describe pain-related neural activation irrespective of learning aspects using a one-sample t-test (Table 1). Results revealed significant neural activation in bilateral insular cortices, bilateral somatosensory cortices (SI, SII) and left VLPFC (ROI analysis: all p_FWE_<.05) and bilateral middle frontal gyri (whole-brain analysis: p<.001 uncorrected) confirming previous imaging findings using rectal distensions [28]–[31].

**Table 1:** Rectal-pain induced neural activation.

Brain regions	MNI Coordinates				
	*H*	*X*	*y*	*z*	*t-value*
**Insula**	**L**	**−34**	**0**	**12**	**7.71** *
	L	−32	8	2	4.29
	R	42	10	−6	4.18
	R	32	8	8	4.91
**Primary somatosensory cortex (SI)**	**L**	**−62**	**−34**	**32**	**6.20** *
	**L**	**−62**	**−24**	**26**	**4.88** *
	**R**	**62**	**−28**	**40**	**5.87** *
**Secondary somatosensory cortex (SII)**	**L**	**−50**	**−32**	**26**	**4.41** *
	**R**	**60**	**−38**	**48**	**5.40** *
	**R**	**62**	**−24**	**30**	**4.65** *
**Prefontal cortex (VLPFC)**	**L**	**−48**	**0**	**10**	**6.42** *
Prefrontal cortex (middle frontal gyrus)	L	−24	34	12	4.14
	L	−38	44	32	3.96
	R	44	50	14	4.59

One-sample test assessing cued rectal-pain induced neural activation, computed on all N = 12 distensions delivered in the acquisition phase using one-sample t-test. Results of whole-brain analysis (p<.001 uncorrected).

* Results that reached significance in ROI analyses with small-volume correction (familywise error correction (FWE); all p_FWE_<.05 in bold print).

**H**, Hemisphere with activation; **R**, right asymmetrical activation; **L**, left asymmetrical activation; **VLPFC**, ventrolateral prefrontal cortex.

### VAS online ratings

CS-US contingency ratings were accomplished separately for CS^+^ and CS^−^ following each phase (i.e., a total of three ratings). The repeated measures ANOVA with two repeated factors (type of CS; time) revealed significant main effects for type of CS (F = 65.53, p<.001), for time (F = 28.38, p<.001) and a significant interaction (F = 45.50, p<.001). Post-hoc comparisons of means (i.e., CS^+^ vs. CS^−^) for each time point revealed significantly greater perceived CS^+^-US contingency following the acquisition phase (p<.001, Fig. 2A). There were no differences in contingency ratings following the extinction and reinstatement phases (Fig. 2A), supporting that the different contingencies were perceived rather correctly by participants, i.e., 75% CS^+^-US reinforcement in the acquisition phase, 0% in the extinction and reinstatement phases.

Pleasantness/unpleasantness ratings were accomplished separately for CS^+^ and CS^−^ at baseline and following each phase, i.e., a total of four ratings. The repeated measures ANOVA revealed a significant main effect for type of CS (F = 22.87, p<.001) and a significant interaction effect (F = 14.12, p<.001). Post-hoc comparisons of means (i.e., CS^+^ vs. CS^−^) for each time point revealed significantly increased CS^+^ unpleasantness following the acquisition phase (p = .001) as well as following the extinction phase (p = .006, Fig. 2B) indicating that this conditioning paradigm evoked negative emotions in response to the CS^+^ as formerly neutral stimulus.

### Brain activation in ROIs during early and late acquisition

**Figure 3:**
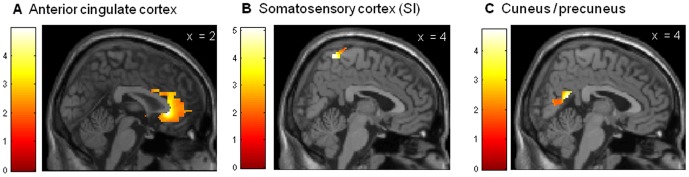
Neural activation within regions-of-interest during early acquisition {CS^+^>CS^−^}. Neural activation during anticipatory conditioned responses in the early acquisition phase assessed within ROIs with a one-sample t-test on the contrast CS^+^>CS^−^ with increase in CS^+^ unpleasantness as covariate of interest. During early acquisition, presentation of the CS^+^ led to significantly greater anticipatory activation in the anterior cingulate cortex (Fig. 3A), somatosensory cortex (Fig. 3B), and the cuneus/precuneus (Fig. 3C) when compared to the CS^−^. All p_FWE_<.05, for more details, see Table 2. Activations were overlaid on a structural T_1−_weighted MRI used for spatial normalization and thresholded at p<.01 for visualization purposes using anatomical templates.

**Figure 4:**
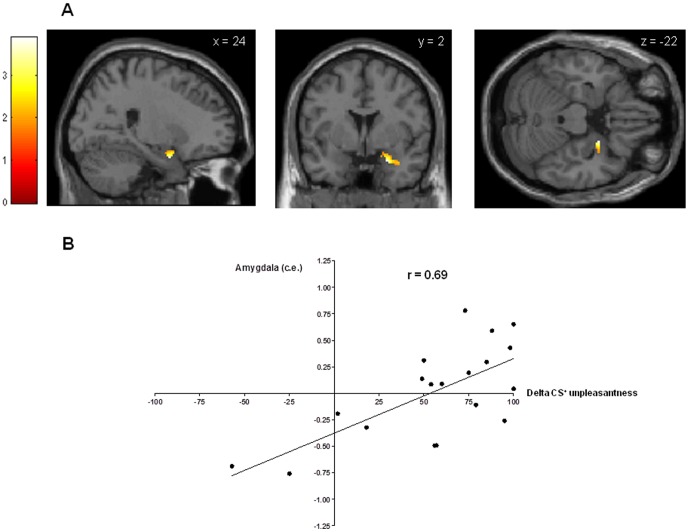
Neural activation within amygdala during late acquisition {CS^+^>CS^−^} and correlation to CS+ unpleasantness. Conditioned anticipatory activation within the amygdala in the late acquisition phase assessed with a one-sample t-test on the contrast CS^+^>CS^−^ with increase in CS^+^ unpleasantness as covariate of interest (p_FWE_<.05), overlaid on a structural T_1−_weighted MRI used for spatial normalization and thresholded at p<.05 for visualization purposes using a bilateral anatomical amygdala template (A). Peak amygdala activation (c.e. = contrast estimates) correlated significantly with the increase in CS^+^ unpleasantness from baseline to the conclusion of the acquisition phase (delta unpleasantness) (Pearson's r = .69, p<.01, B).

Anticipatory conditioned responses in the early and late acquisition phase were analyzed with one-sample t-tests on the contrast CS^+^>CS^−^ correlating with increase in CS^+^ unpleasantness. During early acquisition, presentation of the CS^+^ led to significantly greater anticipatory activation in the somatosensory cortex (Fig. 3A), the anterior cingulate cortex (Fig. 3B) and the precuneus (Fig. 3C) when compared to the CS^−^ (CS^+^>CS^−^: ROI analyses: all p_FWE_<.05, Table 2). Additional whole-brain analyses on the same contrast revealed significant activation of temporal regions (i.e., superior, inferior and parahippocampal gyri), putamen, occipital regions (i.e., fusiform and lingual gyri, cuneus), and cerebellum (all p<.001 uncorrected, Table 2). During late acquisition, significant activation of the right amygdala was found (CS^+^>CS^−^: ROI analysis: p_FWE_<.05, Fig. 4A). Peak amygdala activation correlated significantly with the increase in CS^+^ unpleasantness from baseline to the conclusion of the acquisition phase (r = .69, p<.01, Fig. 4B).

**Table 2:** Anticipatory conditioned responses in the early and late acquisition phase (CS^+^>CS^−^).

Brain Regions	MNI coordinates									
	H	x	y	z	t-value	H	x	y	z	t-value
	Early acquisition (CS^+^>CS^−^)					Late acquisition (CS^+^>CS^−^)				
**Somatosensory cortex (SI)**	**R**	**4**	**−52**	**70**	**5.08** *	**-**	**-**	**-**	**-**	-
**Anterior cingulate cortex**	**R**	**2**	**30**	**−2**	**4.95** *	**-**	**-**	**-**	**-**	-
	L	−2	28	−2	4.44	**-**	**-**	**-**	**-**	-
**Precuneus**	**R**	**4**	**−54**	**18**	**4.71** *	**-**	**-**	**-**	**-**	-
Cuneus	R	4	−90	16	5.09	**-**	**-**	**-**	**-**	-
Temporal lobe (superior temporal gyrus)	L	−32	12	−28	4.50	**-**	**-**	**-**	**-**	-
	R	50	−14	6	4.25	**-**	**-**	**-**	**-**	-
Temporal lobe (Inferior temporal gyrus)	R	42	−6	−20	5.1	**-**	**-**	**-**	**-**	-
	R	44	−28	−14	4.63	**-**	**-**	**-**	**-**	-
Temporal lobe (parahippocampal gyrus)	R	28	6	−12	4.74	**-**	**-**	**-**	**-**	-
Putamen	L	−28	−24	6	4.42	**-**	**-**	**-**	**-**	-
	R	26	8	−10	4.34	**-**	**-**	**-**	**-**	-
Occipital lobe (fusiform gyrus)	R	22	−70	−12	6.46	-	**-**	**-**	**-**	-
	L	−40	−46	−24	4.22	**-**	**-**	**-**	**-**	-
	L	−28	−62	−16	4.22	**-**	**-**	**-**	**-**	-
Occipital lobe (lingual gyrus)	R	20	−70	−12	6.47	**-**	**-**	**-**	**-**	-
Cerebellum	R	16	−68	−12	5.34	**-**	**-**	**-**	**-**	-
	R	32	−66	−18	4.63	**-**	**-**	**-**	**-**	-
	L	−18	−50	−16	4.37	**-**	**-**	**-**	**-**	-
	L	−20	−76	−20	4.27	**-**	**-**	**-**	**-**	-
	L	−38	−50	−26	4.25	**-**	**-**	**-**	**-**	-
	R	6	−72	−10	4.20	**-**	**-**	**-**	**-**	-
	L	−22	−50	−26	4.19	**-**	**-**	**-**	**-**	-
**Amygdala**						**R**	**24**	**2**	**−22**	**3.89** *

Anticipatory conditioned responses in the early and late acquisition phase analyzed with one-sample t-tests on the contrast CS^+^>CS^−^ correlating with increase in CS^+^ unpleasantness. Results of whole-brain analysis (p<.001 uncorrected).

* Results that reached significance in ROI analyses with small-volume correction (familywise error correction (FWE); all p_FWE_<.05 in bold print). The opposite contrast CS^+^<CS^−^ revealed an activation of the right superior temporal gyrus (not shown in the table: x = 46, y = 14, z = −24, t = 4.01, whole-brain analysis: p<.001, uncorrected).

**H**, Hemisphere with activation; **R**, right asymmetrical activation; **L**, left asymmetrical activation.

The opposite contrast (CS^+^<CS^−^) revealed no significant activations in ROI analyses. Exploratory whole-brain analyses demonstrated significant activation of the right superior temporal gyrus (x = 46, y = 14, z = −24, t = 4.01, p<.001, uncorrected).

### Complementary analyses of amygdala activation

**Figure 5:**
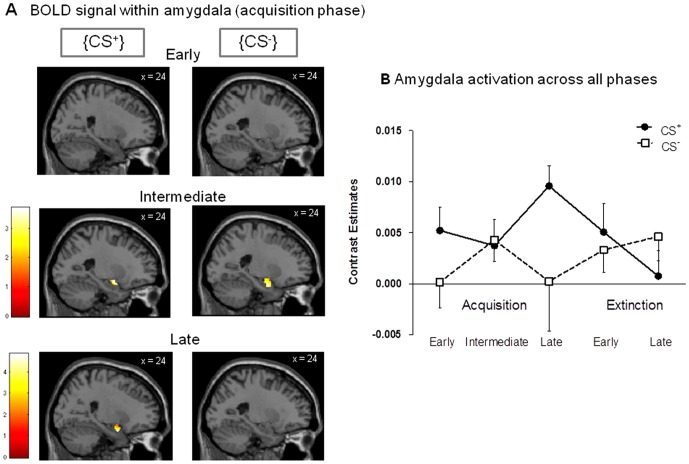
BOLD response within amygdala. Complementary analyses of amygdala responses to the CS^+^ and the CS^−^ over the course of the learning and extinction processes. (A) Separate one-sample t-tests contrasting CS^+^ and CS^−^ were computed for early (top row), intermediate (intermediate row) and late (bottom row) acquisition phases, overlaid on a structural T_1−_weighted MRI used for spatial normalization and thresholded at p<.05 for visualization purposes using a bilateral anatomical amygdala template. (B) Contrast estimates for amygdala activation were extracted, averaged, and plotted for visualization purposes for all phases of the experiment.

For a more refined understanding of amygdala responses to the CS^+^ and the CS^−^ over the course of the learning and extinction processes, we computed separate one-sample t-tests on the CS^+^ and CS^−^ for early (Fig. 5A, top row), intermediate (Fig. 5A, intermediate row) and late (Fig. 5A, bottom row) acquisition phases. This analysis demonstrated that no significant amygdala activation was present during early acquisition in response to either CS, whereas both CS^+^ and CS^−^ evoked amygdala activation in the intermediate acquisition phase. Only during late acquisition there was a clear differentiation between the CS^+^ and the CS^−^ with respect to amygdala activation supporting the establishment of a conditioned fear response at the neural level. Finally, for a better visualization of amygdala activation, we extracted, averaged, and plotted contrast estimates for all phases of the experiment. This further confirmed CS^+^-evoked amygdala activation during late acquisition which quickly and effectively extinguished following unpaired CS^+^ presentations during the extinction phase (Fig. 5B).

### Brain activation in ROIs during extinction & reinstatement

ROI analyses revealed no significant activation in the contrast CS^+^>CS^−^. However, at a more liberal threshold, we found significant activation of the ACC, precuneus, temporal regions (middle temporal gyrus, hippocampus), putamen, insula and middle frontal gyrus (whole-brain analysis: all p<.001 uncorrected, Table 3 left columns). The opposite contrast CS^−^>CS^+^ revealed significant activation of the middle frontal gyrus (ROI analysis: p_FWE_<.05), and additionally at a more liberal threshold activation of other prefrontal regions (i.e., superior and medial frontal gyri, whole-brain analyses, p<.001, Table 3 right columns).

**Table 3:** Anticipatory conditioned responses during extinction.

Brain Regions	MNI coordinates									
	H	x	y	z	t-value	H	x	y	z	t-value
	CS^+^>CS^−^					(CS^−^>CS^+^)				
Anterior cingulate cortex	R	12	24	26	4.14					
Precuneus/cuneus	R	26	−66	26	5.58					
	L	−18	−80	50	4.92	L	−6	−58	34	4.17
	L	−26	−70	40	4.87					
Temporal lobe (middle temporal gyrus)	L	−48	−46	6	4.28					
Temporal lobe (hippocampus)	L	−28	−36	0	4.21					
Putamen	L	−22	12	10	3.9					
Insula	L	−40	−24	22	3.91					
**Prefrontal cortex (middle frontal gyrus)**	R	32	6	46	4.44	**R**	**26**	**12**	**46**	**5.28** *
	L	−20	36	−16	4.05					
Prefrontal cortex (superior frontal gyrus)						L	−6	58	40	4.21
Prefrontal cortex (medial frontal gyrus)						L	−10	48	22	4.2
						R	4	54	46	4.01
						R	4	42	24	4.17

Anticipatory conditioned responses in the early extinction phase with one-sample t-tests on the contrast CS^+^>CS^−^ and the opposite contrast CS^−^>CS^+^ correlating with decrease in CS^+^ unpleasantness. No significant activations were observed for the late extinction phase.

Results of whole-brain analysis (p<.001 uncorrected).

* Results that reached significance in ROI analyses with small-volume correction (familywise error correction (FWE); p_FWE_<.05 in bold print). **H**, Hemisphere with activation; **R**, right asymmetrical activation; **L**, left asymmetrical activation.

During reinstatement, activation of the parahippocampal gyrus (x = −32, y = −32, z = −4, t = 3.93) was observed in the contrast CS^+^>CS^−^ in a whole-brain analysis (with a threshold of p<.001), but this activation did not reach significance in ROI analysis (p_FWE_ = .09).

**Figure 6:**
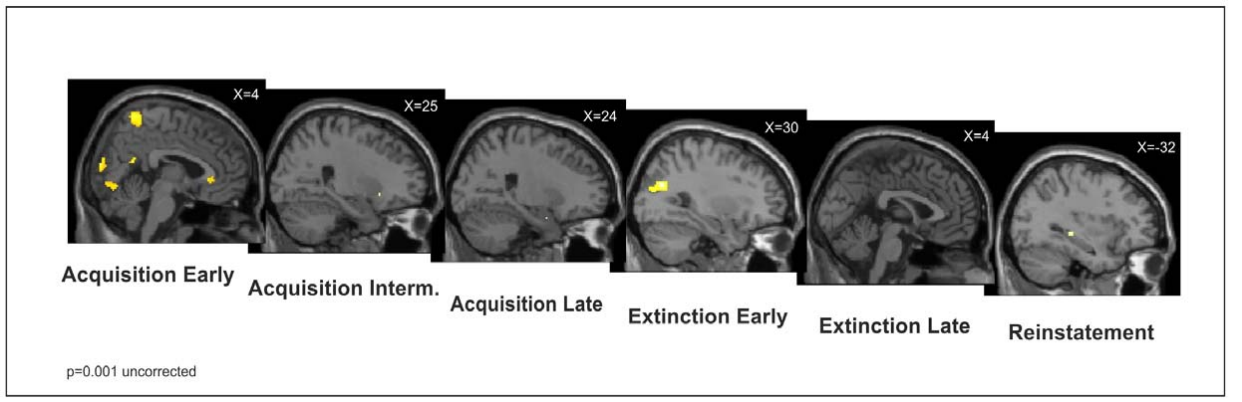
Series of whole-brain images for all phases of the study. For illustration purposes, activations found in a whole-brain analysis (p<.001, uncorrected) during early, intermediate and late acquisition, early and late extinction, and reinstatement were overlaid on a structural T_1−_weighted MRI used for spatial normalization using anatomical templates.

For illustration purposes, Figure 6 shows a series of whole-brain images for all phases of the study.

## Discussion

We implemented the first Pavlovian conditioning study in which the conditioned anticipatory brain responses to painful rectal pain stimuli as US were analyzed with fMRI in healthy subjects during associative learning, extinction and reinstatement. In summary, differential conditioning with rectal US is feasible and leads to learned unpleasantness of previously neutral predictive stimuli. Within the brain, core areas of the central fear network including the amygdala and the anterior cingulate cortex together with somatosensory cortex and precuneus are activated during associative learning. During extinction, conditioned responses quickly disappear, and learning of new predictive cue properties is paralleled by prefrontal activation. Finally, it may be possible to show reactivation of the old memory trace during reinstatement. Together, these findings contribute to our understanding of aversive visceral learning and memory processes relevant to the pathophysiology and treatment of chronic abdominal pain.

### The associative learning process in aversive visceral conditioning

The learned association between visual CS^+^ and painful rectal US resulted in contingency awareness and a valence change, i.e., significantly increased unpleasantness of a previously neutral predictive stimulus. Within the brain, these subjective changes were paralleled by increased anticipatory activation of the anterior cingulate cortex, the somatosensory cortices and precuneus in the early acquisition phase, and of the amygdala in the late acquisition phase. The amygdala constitutes a crucial structure in fear conditioning [1], [32]. Our results extend existing findings with other types of (usually much shorter) US by demonstrating for the first time that the amygdala is also activated in a differential fear conditioning paradigm involving (relatively longer) painful rectal distensions as US. Our supplementary analyses provided a more refined understanding of amygdala activation across the associative learning process. While no amygdala activation was present during early acquisition in response to either CS, interestingly both CS^+^ and CS^−^ evoked amygdala activation in the intermediate acquisition phase which could reflect the degree of uncertainty in our paradigm given the 75% reinforcement schedule. Only during late acquisition there was a clear differentiation between the CS^+^ and the CS^−^ with respect to amygdala activation, and correlational analysis supported that individuals who demonstrated a strong increase in CS^+^ unpleasantness during fear conditioning also showed greater amygdala activation. Of note, our finding of late amygdala activation is at odds with some previous work demonstrating amygdala activation in the early phase of fear acquisition. However, most of these studies utilized a continuous (i.e., 100%) CS^+^-US pairing schedule [33]–[36]. Clearly, the time course of neural responses and the degree/extent of activation may be influenced by contingency. In partial reinforcement designs, such as ours, US expectancy is decreased which slows conditioning [37]–[39]. Hence, our partial reinforcement design may explain the amygdala activation in the late acquisition phase.

The anterior cingulate cortex, the somatosensory cortices and precuneus were also activated during the associative learning process. However, these regions were only activated during early acquisition, indicating that they may mediate learning aspects distinct from those of the amygdala. As for the ACC, it has recently been proposed that the cingulate cortex mediates *potential* threat assessment, whereas the amygdala is involved in *immediate* fear-provoking threats [40]. This distinction fits with our results. Accordingly, during early learning ACC activation may reflect the process of potential threat evaluation while the later amygdala activation may reflect learned anticipatory responses to immediate fear-provoking cues. Our ACC result further extends previous evidence about the role of the ACC in the context of visceral pain and negative emotions (reviewed in [18]). Via connections to the amygdala, the ACC is part of the central network responsible for the coordination of affective reactions to painful stimuli by encoding emotional, motivational and cognitive demands [41]–[43]. Fear and pain sites reportedly overlap in the ACC, which is therefore thought to specifically mediate the *fear avoidance* aspect of pain processing [44]. Hence, we conclude that the ACC plays a crucial role in the early associative learning process, together with somatosensory areas and the precuneus which fits its established role in memory and visuospatial perception.

Our findings are consistent with previous aversive delay conditioning studies using other unconditioned stimuli (e.g., electric shock, reviewed in [1]), and complement and extend the only existing previous visceral aversive conditioning fMRI study with gastrointestinal US (i.e., esophageal distensions) [6]. The authors found that presentation of the CS alone led to activation of a number of brain regions that were also seen during actual esophageal distensions. We could now show that aversive visceral conditioning is also feasible with rectal stimuli, and that conditioned anticipatory amygdala activation correlates with learned unpleasantness of a previously neutral CS in healthy subjects. Similar to our study, part of their analyses were aimed at elucidating anticipatory activation irrespective of US-induced neural activation, however this was accomplished following completion of a learning phase. Hence, findings concerning neural activation during the acquisition of anticipatory conditioned responses cannot be directly compared. Nevertheless, the authors' conclusion that the anticipation of pain deserves further attention is underscored by our results. Several recent studies have made an effort to elucidate the role of anticipation in human visceral pain [28], [29], [45]–[49] and recently in an animal model assessing retrieval of visceral pain-conditioned passive avoidance [8]. In existing human studies, however, possible learning (and memory) aspects on anticipation have been largely neglected. Clearly, more studies are urgently needed to assess the putative clinical relevance of associative learning phenomena as a tool to understand the role of pain anticipation and pain memory.

### Extinction & Reinstatement

Extinction is not simple “forgetting” or “erasing” of an old memory, but rather a form of new inhibitory learning that is different than the initial acquisition of the CS-US-association [19]. Little is known regarding extinction processes in the context of (chronic) pain in general, with the exception of highly interesting findings suggesting impaired extinction in chronic low back pain [50], [51] and persisting hypervigilance towards pain signals even after extinction in healthy subjects [52]. On the other hand, extinction deficits have been well-documented in fear conditioning paradigms in various anxiety disorders [16], and recently a treatment protocol involving exposure therapy has been successfully implemented in IBS [53]. In their conditioning study using esophageal USs, Yágüez et al. observed that during extinction, the CS^+^ continued to elicit neural activation similar to the response seen during painful stimulation, i.e., ACC, somatosensory cortex and insula, albeit with markedly reduced spatial extent and intensity of activation [6]. We found activation of the middle frontal gyrus, and (at a lower level of statistical significance) of the precuneus, ACC and temporal regions including the hippocampus during the early extinction phase, similar to the activation seen during early acquisition. This pattern of activation, however, was somewhat different from that seen during actual rectal distensions, suggesting that new, inhibitory learning of predictive cue properties may not necessarily involve identical structures as those relevant for US processing. During late extinction, we could no longer detect any significant activations (not even in whole-brain analysis at p<.001 uncorrected), supporting that extinction was “complete” and hence took place following relatively few unpaired CS presentations. This relatively fast extinction process could be explained by the choice of a delay (over a trace conditioning) protocol which reportedly leads not only to more rapid learning of CS-US association in the acquisition phase as well as to fast(er) extinction [1]. Interestingly, Yágüez et al. also reported additional activation of the DLPFC and the supplementary motor area [6] during extinction in their study, which is consistent with our finding of prefrontal activation (i.e. middle, superior, medial prefrontal gyri) in both contrasts CS^+^>CS^−^ and CS^−^>CS^+^. These findings likely indicate a new learning process regarding predictive cue properties during extinction, involving higher cognitive functioning mediated by prefrontal cortices.

Extinguished responses do not necessarily disappear but can return spontaneously (“spontaneous recovery”) or following manipulations such as reinstatement or renewal [54]. Although reinstatement has been studied in humans in the context of fear conditioning (reviewed in [16]), only few fMRI studies exist addressing the putative neural mechanisms. Our analysis of anticipatory responses to conditioned stimuli, i.e., CS^+^>CS^−^, that followed the single, unpaired US revealed activation of the parahippocampal region in a whole-brain analysis at p<.001 uncorrected. While this is consistent with our hypothesis and suggests possible reactivation of the memory trace, this finding has to be viewed with caution since it did not reach statistical significance in ROI analysis.

### Limitations & Conclusions

Our study did not include other well-characterized objective peripheral markers of conditioning such skin conductance recordings, which is one important limitation. In addition, the potential role of sensitization processes over the course of repeated stimulus presentations needs to be clarified in future studies that should consider including (a) control group(s) exposed to identical stimuli but in a non-paired manner. Nevertheless, our study is one of the first in the field of visceral pain to support the putative role of predictive external cues, i.e., visual stimuli, in shaping anticipatory responses to aversive visceral stimuli. Cleary, in “real life” situations, numerous putative cues predicting aversive visceral signals are present, including internal as well as external cues or contexts (e.g., specific foods, smells, situations). For example, is has been suggested that bodily sensations can turn into predictors of pain and through interoceptive fear conditioning start eliciting a fear response [5]. Given recent suggestion that fear extinction constitutes a model for translational behavioral neuroscience [2] and the clinical relevance of extinction training and exposure therapy in anxiety disorders [55], more translational knowledge about associative learning and extinction in visceral pain could contribute to new cognitive-behavioral treatment options in IBS [49], [56], including exposure therapy [53].
